# The Dissociable Effects of Induced Positive and Negative Moods on Cognitive Flexibility

**DOI:** 10.1038/s41598-018-37683-4

**Published:** 2019-02-04

**Authors:** Shulan Hsieh, Siang Jyun Lin

**Affiliations:** 1 Department of Psychology, National Cheng Kung University, Tainan City, Taiwan; 2 Institute of Allied Health Sciences, National Cheng Kung University, Tainan City, Taiwan; 3 Institute of Behavioral Medicine, National Cheng Kung University, Tainan City, Taiwan

## Abstract

This study investigates how different valences of induced moods modulate cognitive flexibility in a task-switching paradigm. Forty-eight participants aged 19–25 years performed task switching after watching emotional film clips to induce an emotion (neutral, positive, or negative emotions). Two indicators of flexibility were evaluated: (1) the performance decrement reflected by increased reaction time (RT) or errors on the task-switch trial relative to a task-repetition trial, which is known as the “switching cost,” and (2) the performance improvement reflected by decreased RT or errors when switching from a task-switching context to a single-task context, which is known as the “fade-out” effect. These indicators reflect cognitive flexibility on short and long time scales, respectively. The results show that negative moods reduced switching costs, particularly in incongruent trials. In addition, negative moods were found to cause a prolonged fade-out effect compared with neutral and positive moods, indicating that participants required more trials to adjust to the single-task condition after experiencing the task-switching context. The result suggests that only negative moods and not positive moods modulated both the short and long time scales of cognitive flexibility but with dissociable effects. Possible explanations are discussed.

## Introduction

This study examines the effect of induced moods on cognitive flexibility using emotional video clips, particularly in regard to whether positive and negative emotional valences modulate the effect and how. Research has identified close relationships that emotion and mood have with cognition^1–14^. For example, recent studies indicate that people with emotional problems have cognitive impairments^1–4^. In addition, studies have shown that emotion or mood modulates various cognitive functions, including selective attention^5^, response inhibition^6^, categorization^7^, creative problem solving^8,9^, attentional control^10^, cognitive flexibility^11,12^, and self-control or self-regulation processes^13,14^.

Among these functions, this study focuses on cognitive flexibility as measured by the task-switching paradigm^15–17^. Task switching is an experimental paradigm that is used to explore the mechanisms of cognitive control that enable human behavioral flexibility in the fields of cognitive neuroscience^18^, aging^19^, clinical science^20^, and educational research^21^. Various forms of the task-switching paradigm have been developed^22,23^. In the conventional cueing task-switching paradigm, participants are required to switch rapidly between two tasks, which are indicated by a task cue that is presented prior to a target stimulus, or to repeat the same task as in the preceding trial in a mixed-task block. The switching cost is measured as an indicator of rigidity, which is calculated by subtracting the reaction time (RT) or the percentage of error (PE) of a repeat trial from that obtained in a switching trial with a mixed-task block. This paradigm has been widely used to study cognitive flexibility to evaluate the ability to adapt rapidly and momentarily, as well as to anticipate, select, prepare, and implement action plans efficiently^22,23^.

The task-switching paradigm involves multiple distinct processing factors. For example, in addition to the switching cost, the impact of the global context can also be measured using the difference in RT between repeat trials derived from mixed-task blocks and single trials derived from single-task blocks, which is known as the “mixing cost.” This cost has been hypothesized to reflect the strategic processes that participants use to maintain a switching mode of remaining prepared for both task sets throughout a block, irrespective of whether or not changing tasks is actually necessary^16^.

To test this hypothesis, Mayr and Liebscher^24^ developed a variant of the task-switching paradigm known as the “fade-out” task-switching paradigm, in which remaining prepared for both task sets throughout a block is not a rational strategy. Participants are instructed to perform the tasks in a task-switching mode with a mixed-task block, in which both switched tasks are relevant and intermixed. They are then instructed to shift back to a single-task mode with a purely single-task block, where only one of the two tasks is relevant throughout the block. These subsequent single-task blocks are called “fade-out” blocks, and participants are explicitly cued on a trial-to-trial basis to respond to only one of the tasks. Therefore, under these circumstances, it is no longer a rational strategy for participants to remain prepared for two task sets.

The fade-out effect refers to the improvement in performance when moving from a switching mode to single-task mode, which is reflected by the trend of how the RTs in the trials become shorter in the course of the fade-out block. These changes in RT can be observed as a function of each trial or each segment in the fade-out block by pooling over the performance of several trials (e.g., 10, 16, 20, or even 40 trials, depending on the total number of trials of a block). This fade-out effect reflects the rate of disengagement from a switching mode. Thus, the effect also indicates “rigidity” because it shows whether the person still maintains readiness for task switching even though they have already changed to the single-task context where task switching is no longer required^24^.

The advantage of the fade-out task-switching paradigm is that it provides two time scales of rigidity indicators: a short time scale through the switching cost, which reflects the transition cost between two switched tasks, and a long time scale through the fade-out cost, which reflects the transition cost between the two processing modes. The fade-out costs can be calculated by subtracting the RT or the PE of a single-task block preceding a mixed-task block from that of each trial or segment of the subsequent fade-out block. The fade-out effect has been used to investigate differences in cognitive flexibility among elderly adults^24,25^ and people with unipolar depression or obsessive-compulsive disorder^26^. The paradigm is highly ecologically valid since cognitive flexibility refers to both momentary shifting and control-mode shifting on a long time scale^16,24,26^.

Compared with other types of executive function, few studies have directly investigated the effect of emotional valence on the task-switching paradigm. In addition, these studies focused on only a short time scale of rigidity (i.e., switching costs)^11,27–31^. None of the studies have investigated the effect of emotional valence on a long time scale of rigidity (i.e., fade-out costs). Therefore, the purpose of this study is to gather more empirical evidence about the effect of emotional valence on flexibility by means of the fade-out task-switching paradigm.

Currently, there is no single theory that can directly predict the effect of emotional valences on different time scales of cognitive flexibility. Nevertheless, we borrowed from some available theories and combined them to make indirect predictions. For example, researchers have shown that negative emotion can facilitate cognitive and emotional conflict processing^32,33^. Negative emotion seems to narrow the scope of attention^34,35^, which means it may facilitate the processing of conflict by either amplifying the processing of the target dimension or reducing the influence of emotional distractors that are irrelevant to the task^33^. This view is also in line with the suggestion that the human brain is equipped with a *fear module* that promotes the modulation of selective attention toward evolutionarily threating stimuli, hence facilitating cognitive control^36,37^.

Accordingly, we hypothesize that negative moods reduce switching costs. However, it is unclear whether negative moods would facilitate or impede the fade-out effect since fade-out costs differ from switching costs in that they involve the rate of disengagement from a control-demand mode (switching mode) to less of a control-demand mode (single-task mode). Negative moods may impede the relaxation of cognitive control. Meiran and colleagues provide supporting evidence through observations that patients with unipolar depression or obsessive-compulsive disorder had increased fade-out costs^26^. This suggests that these patients with negative moods required more trials to adjust to single-task blocks after experiencing the task-switching mode. In a similar vein, we predicted that negative moods would result in larger fade-out costs. Alternatively, updating working memory is required to flexibly switch task goals, which places demands on cognitive capacity^16^. As such, negative moods may deplete cognitive resources and reduce the efficiency of updating processing modes, resulting in larger fade-out costs.

Some researchers suggest that positive emotion could also facilitate conflict processing, executive control, and creative problem solving^9,32,33,38,39^. For example, Kanske and Kotz^38^ and Zinchenko *et al*.^39^ showed that both positive emotions and novelty in demanding task conditions facilitate conflict processing and executive control. Subramaniam and colleagues^9^ suggested that positive moods enhance insightful solutions for solving problems, possibly by modulating attention and cognitive control mechanisms via the anterior cingulate cortex (ACC) to facilitate more sensitivity to detect competing solution candidates, which is a form of cognitive flexibility.

Accordingly, we hypothesized that positive emotions would reduce switching costs and possibly also fade-out costs^11,30^. This view is in line with *appraisal theories*, which suggest a general mechanism in which a central determinant of emotion is the rapid, multilevel assessment of the relevance of a stimulus to the goals, needs, and wellbeing of an individual, independent of the valence of the stimulus^40,41^. Based on these hypotheses, if both positive and negative emotional valences involve similar mechanisms in modulating cognitive flexibility, then both valences should result in similar effects on both time scales of cognitive flexibility. Conversely, if positive and negative emotional valences involve differential mechanisms, then only one of the emotional valences would modulate one of the time scales.

We also manipulated two other variables in addition to the different time scales. One variable is the task preparation time, which was investigated by using two randomly determined cue-target intervals (CTIs): 100 ms (which affords little or no preparation) and 1000 ms (which provides a long time for preparation). In the critical fade-out block, an explicit cue is provided in advance to indicate which of the tasks is the only relevant one throughout the single-task block. Switching cost can be reduced when participants are provided more time through longer CTIs to prepare for a task switch or to disengage from the preceding task set^17,42,43^.

Compared to the switching cost, the mixing cost and fade-out cost have received less attention regarding whether CTIs modulate their magnitudes^43^. Nevertheless, one study on fade-out has shown that different CTIs can modulate the magnitude of age differences in fade-out costs^24^. Therefore, we expect that longer CTIs might yield more time for participants to disengage from the task-switching mode and transition to the pure single-task mode and that it might further modulate the effect of emotion on the fade-out cost.

The other variable is the congruency effect. In a typical task-switching experiment, participants classify multidimensional stimuli according to a particular task rule. These task rules typically map dimensional values such as *red* or *green* (a color dimension) onto response keys such as *right* or *left* (or vice versa). In a task-switching experiment, a relevant task rule dictates the correct response that should be chosen for each trial. However, there is also an irrelevant task rule (e.g., a shape dimension), which is related to another task and associated with another set of responses that was required in the past or will be required in the future (e.g., a *triangle* mapped to the *right* response key and a *square* mapped to the *left* response key, or vice versa). Therefore, irrelevant task rules might activate either a *congruent* response or an *incongruent* response. For example, task rules for color and shape dimensions related to a *red triangle* stimulus could potentially activate the same response key (e.g., *right*), which is known as *congruent* trials. However, tasks rules for color and shape dimensions related to a *red square* stimulus could potentially activate two competing response keys (e.g., *right* vs. *left*), which is known as *incongruent* trials.

The congruency effect refers to the relatively poor performance of a participant on incongruent trials in which the irrelevant task rule activates a competing response to that in congruent trials, in which the irrelevant rule activates the same response. The congruency effect is an important behavioral marker for the costs associated with maintaining task readiness (see Sudevan and Taylor^44^ for the first demonstration of this marker and Meiran and Kessler^45^ for a review). Therefore, we suspected that the emotional valence effect on switching costs might further interact with the congruency effect.

## Results

### Subjective Emotion Rating Scales

The subjective emotional responses to the film clips (i.e., the scores on the emotion category scale) confirmed the original normative data showing that these films successfully induced pure emotions, including amusing, sad, and neutral emotions. The 9-point Likert scale’s mean score of the targeted emotion was 4.96 for the amusing film clip, and 4.33 for the sad film clip. In addition, participants’ ratings of the target emotions significantly different from other emotions within each condition (ps < 0.0001). In this study, the overall mean hit rate for the amusing and sad film clips were 87.50% and 93.75%, respectively.

In addition, a repeated-measures one-way analysis of variance (ANOVA) was performed on the valence and arousal SAM dimension scores for the positive, neutral, and negative film clips. As expected, both dimensional analyses yielded significant results with regard to differentiating emotions. For the valence dimension, a significant main effect of valence was found, F (2, 94) = 152.17, p < 0.01. Tukey post hoc analyses showed that the positive film clip (7.44 ± 0.99) induced more pleasant feelings than the neutral (5.15 ± 0.74), q (94, 3) = 15.11, p < 0.01, and negative film clips (3.73 ± 1.22), q (94, 3) = 24.45, p < 0.01, and the negative film clip induced more unpleasant feelings than the neutral film clip, q (94, 3) = 9.34, p < 0.01. Likewise, a significant main effect of emotion was found for the arousal dimension, F (2, 94) = 26.21, MSE = 81.02, p < 0.01. Tukey post hoc analyses showed that both positive (5.29 ± 1.79) and negative (5.33 ± 1.91) emotional film clips induced more arousal than the neutral film clip (3.06 ± 1.77; neutral vs. positive: q (94, 3) = 8.79, p < 0.01; neutral vs. negative: q (94, 3) = 8.95, p < 0.01), and there was no difference in arousal between positive and negative film clips. As for the dominance dimension, a significant main effect of emotion was found to be significant, F (2, 94) = 11.86, p < 0.0001. Tukey post hoc analyses showed that both positive (4.23 ± 2.01) and negative (4.63 ± 2.18) emotional film clips induced more dominance than the neutral film clip (2.83 ± 1.90; neutral vs. positive: q (94, 3) = 4.23, p < 0.01; neutral vs. negative: q (94, 3) = 4.72, p < 0.01).

### Fade-Out and Task-Switching Task Performance

RT and PE were calculated in this study. Before the RT analyses, we excluded trials following an error and replaced RTs greater than 3000 ms with missing values. We primarily focused on the results regarding the effect of emotion on the various rigidity indicators such as switching costs (along with the congruency effect), mixing costs, and fade-out costs. Switching costs refer to the performance differences (RT, PE) between switch and non-switch trials in the mixed-task blocks. The congruency effect refers to the performance differences between congruent and incongruent trials. Mixing costs refer to the performance differences between trials in the single-task blocks and non-switch trials in the mixed-task blocks. To calculate fade-out costs, we first subdivided each 64-trial single-task block and fade-out block into 16-trial segments, resulting in 4 segments per block. Fade-out costs refer to the performance differences between each segment of the single-task block and corresponding segment of the fade-out block (i.e., the block immediately following the mixed-task block).

### Emotion, Congruency, and Switching costs

#### RT

**Figure 1:**
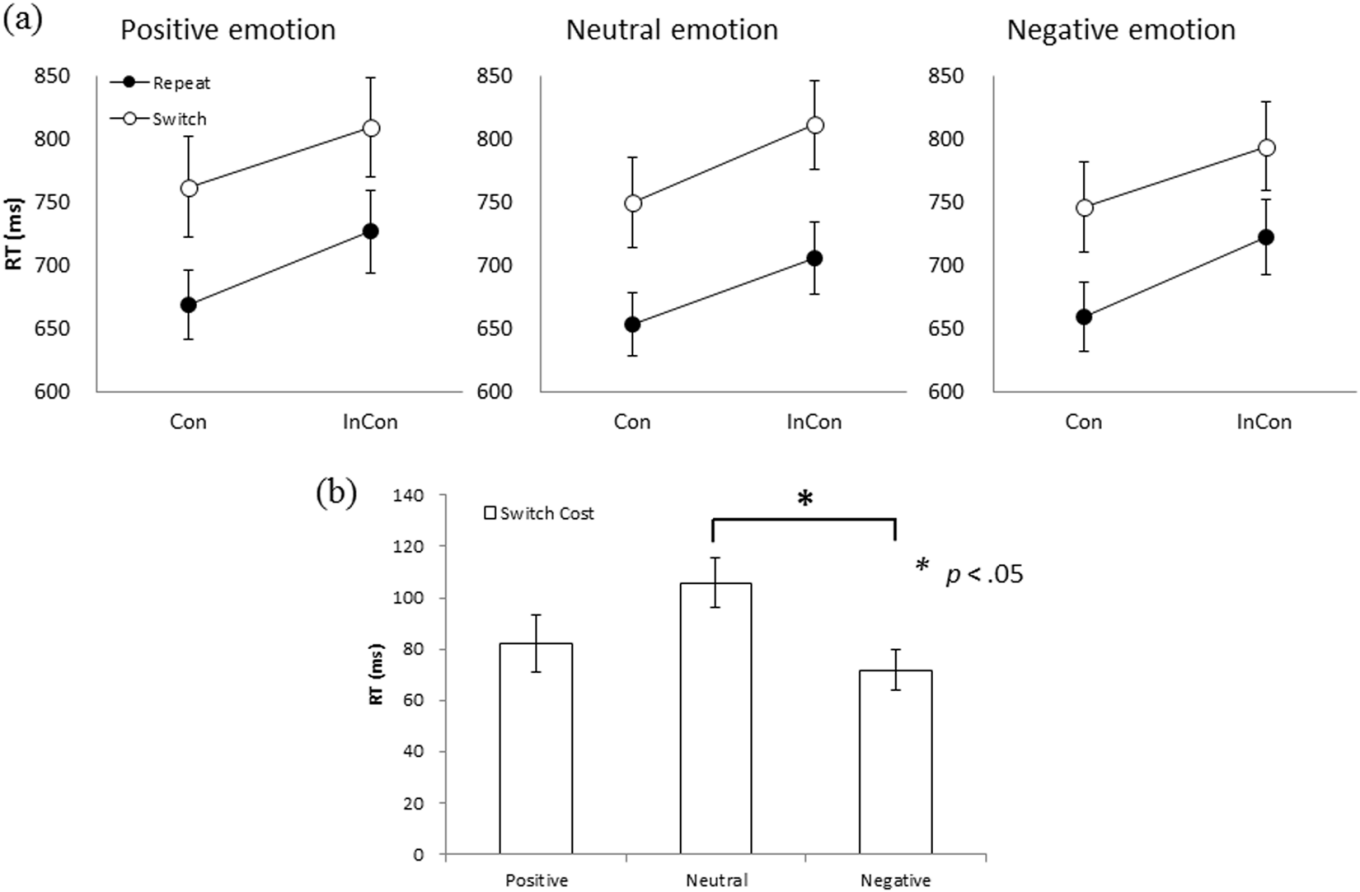
(**a**) Mean RT (ms) as a function of emotion, transition (repeat and switch), and congruency (Con = congruent trials, InCon = incongruent trials); (**b**) Mean switch costs, RT(switch) - RT(repeat), according to three emotion conditions for incongruent-trial conditions only. Error bars represent standard errors. Switching costs for negative emotion were significantly smaller than those for the neutral emotion (p < 0.05).

A 4-way ANOVA on RTs retrieved from the mixed-tasks blocks (for the purpose of calculating switching costs) was performed with emotion (Positive, Neutral, & Negative), transition (Repeat & Switch), CTI (100 ms & 1000 ms), and congruency (Congruent & Incongruent) as within-participant independent variables. The results revealed main effects of transition, *F* (1, 47) = 166.55, *p* < 0.001, showing faster responses for repeat (689 ± 198.11 ms) than switch (779 ± 256.82 ms) trials, of CTI, *F* (1, 47) = 926.47, *p* < 0.001, showing faster responses for 1000-ms CTI conditions (562 ± 115.70 ms) than 100-ms CTI conditions (906 ± 190.43 ms), and of congruency, *F* (1, 47) = 198.30, *p* < 0.001, showing faster responses for congruent conditions (706 ± 228.63 ms) than incongruent condition (762 ± 235.33 ms). Significant 2-way interactions were found between transition and CTI, *F* (1, 47) = 129.88, *p* < 0.001, showing larger switching costs for 100-ms CTI conditions than 1000-ms CTI conditions, and between CTI and congruency, *F* (1, 47) = 6.55, *p* < 0.05, showing larger congruency effect (incongruent–congruent) for 100-ms CTI conditions than 1000-ms CTI conditions. Significant 3-way interactions among emotion, transition and congruency, *F* (2, 94) = 3.55, *p* < 0.05, as well as among transition, CTI and congruency, *F* (2, 94) = 35.52, *p* < 0.001, were also found (see Fig. 1). Additional main effects and interactions were not found.

A simple interaction test was conducted following the 3-way interaction of emotion, transition, and congruency. This test revealed a significant interaction between emotion and transition in the incongruent condition, *F* (2, 139) = 5.11, *p* < 0.01. A subsequent simple main effect test on the incongruent-trial conditions revealed a significant main effect of transition for each emotion condition (positive: *F* (1, 142) = 72.83, *p* < 0.001; neutral: *F* (1, 142) = 119.92, *p* < 0.001; negative: *F* (1, 142) = 55.47, *p* < 0.001).

To further investigate if the switching costs for the three emotion conditions differed significantly among each other, specifically on the incongruent trials, a 1-way ANOVA on switching costs for the incongruent trials was conducted. The results (see Fig. 1b) showed a significant main effect of emotion, *F* (2, 94) = 4.57, *p* < 0.05, in which the switching costs for the negative emotion (71.94 ± 53.52 ms) were significantly smaller than those for the neutral emotion (105.77 ± 65.41 ms; *q*(94, 3) = 4.18, *p* < 0.05).

#### PE

The 4-way ANOVA on PE retrieved from the mixed-tasks blocks (for the purpose of calculating switching costs) revealed significant main effects of emotion, *F* (2, 94) = 4.80, *p* < 0.05, transition, *F* (1, 47) = 35.58, *p* < 0.001, and congruency, *F* (1, 47) = 94.58, *p* < 0.001. The mean PE was higher for neutral emotions (7.80 ± 6.49%) compared with negative emotions (6.28 ± 6.51%), q (94, 3) = 4.25, p < 0.01. There were no differences on PE between positive emotions (7.30 ± 7.10%) and neutral emotions, and between positive emotions and negative emotions. The mean PE was significantly higher for switch trials (7.91 ± 6.90%) compared with repeat trials (6.30 ± 6.40%), and for incongruent-trial conditions (9.20 ± 7.59%) compared with congruent-trial conditions (5.11 ± 4.90%).

**Figure 2:**
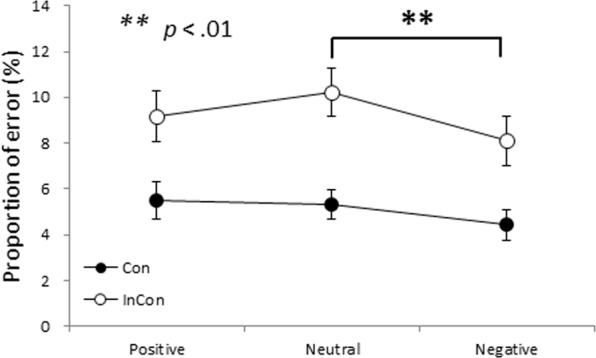
Mean PE (%) as a function of emotion and congruency (Con: congruent trials, InCon: incongruent trials). Error bars represent standard errors. Negative emotions entail less PE than neutral emotions in the incongruent-trial conditions (p < 0.01).

Three significant 2-way interactions were found: transition and CTI (i.e., the effect of CTI was larger for switch than repeat trials; *F* (1, 47) = 35.22, *p* < 0.001), emotion and congruency (i.e., the effect of emotion occurred only for the incongruent-trial conditions; *F* (1, 47) = 3.34, *p* < 0.05), and transition and congruency (i.e., switching costs on PE were larger for congruent-trial than incongruent-trial conditions; *F* (1, 47) = 6.78, *p* < 0.05). In addition, a significant 3-way interaction among transition, CTI, and congruency, *F* (1, 47) = 33.25, *p* < 0.001, was found. No other main effects or interactions were found. With regard to our primary interest, simple effect tests following the significant 2-way interaction of emotion and congruency, *F* (2, 94) = 3.34, *p* < 0.05, indicated that the effect of emotion on PE was significant only for the incongruent-trial conditions, *F* (2, 145) = 6.89, *p < *0.01. For the incongruent-trial conditions, neutral emotions (10.23 ± 7.30%) were associated with significantly more errors than negative emotions (8.12 ± 7.49%; *q* (188, 3) = 5.25, p < 0.01); that is, negative emotions entailed less PE than neutral emotions in the incongruent-trial conditions (see Fig. 2).

### Emotion and Fade-Out Costs

#### RT

A 4-way ANOVA on RT was performed with block (single-task block & fade-out block), CTI (100 ms & 1000 ms), emotion (Positive, Neutral, & Negative), and segment (Segment 1 through Segment 4 and 16 trials for each) as independent variables. The analysis was done on the single-task blocks preceding and following the mixed-task block only, we called single-task block and fade-out block here.

The 4-way ANOVA on RT revealed significant main effects of block, *F* (1, 47) = 55.39, *p* < 0.001, and CTI, *F* (1, 47) = 122.25, *p* < 0.001. The mean RT was significantly faster in single-task blocks (439 ± 67.88 ms) compared with fade-out blocks (474 ± 88.72 ms) and 1000-ms CTI (439 ± 73.38 ms) compared with 100-ms CTI (474 ± 84.37 ms). A significant 4-way interaction was found among block, CTI, emotion, and segment, *F* (6, 282) = 2.84, *p < *0.05. In addition, a significant 3-way interaction was found among block, emotion, and segment, *F* (6, 282) = 2.57, *p* < 0.05. Two significant 2-way interactions were found between block and CTI (i.e., a larger block effect [fade-out vs. single-task block] for 100-ms CTI than 1000-ms CTI conditions; *F* (1, 47) = 7.83, *p* < 0.01), and between CTI and segment (i.e., a larger segment effect for 100-ms CTI than 1000-ms CTI conditions; *F* (1, 47) = 7.49, *p* < 0.001). No other main effects or interactions were found. Only the post hoc analyses following the interactions that involved emotion are reported below.

**Figure 3:**
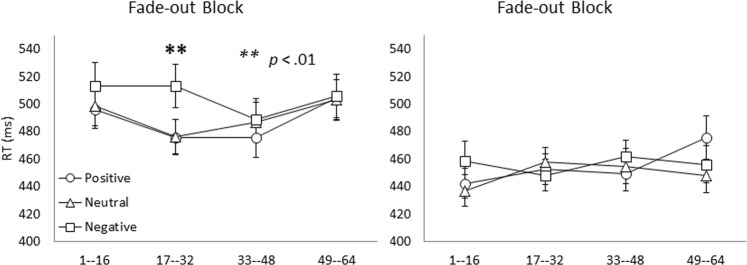
Mean RT according to CTI (left panel: 100 ms-CTI; right panel: 1000 ms-CTI), emotion, and sequential segments (16 trials per segment) within the first fade-out block following the mixed-task block (task switching block). Error bars represent standard errors. The RTs for negative emotions were slower than (*p* < 0.01) those for neutral and positive emotions in Segment 2 (trials #17–32) of the fade-out block for 100-ms CTI conditions.

Post hoc tests following the 4-way interaction of block, CTI, emotion, and segment revealed only one significant 3-way interaction of block, CTI, and emotion for Segment 2, *F* (2, 260) = 6.38, *p* < 0.01, but no such a 3-way interaction significant for other segments. An additional simple interaction test revealed a significant 2-way interaction between block and emotion for the 100-ms CTI in Segment 2, *F* (2, 251) = 6.32, *p* < 0.01, but no such a significant 2-way interaction for the 1000-ms CTI in Segment 2 (p = 0.57). A subsequent simple test revealed a significant main effect of emotion in the fade-out block for the 100-ms CTI in Segment 2, *F* (2, 280) = 5.27, *p* < 0.01, which indicates that the RTs for negative emotions (513 ± 105.32 ms) were slower than those for neutral (477 ± 68.37 ms), *q* (1504, 3) = 3.95, *p < *0.05, and positive emotions (476 ± 82.34 ms), *q* (1504, 3) = 4.00, *p* < 0.05; see Fig. 3. Another post hoc analysis following the significant 4-way interaction of block, CTI, emotion, and segment also revealed a significant 3-way interaction effect of block, emotion, and segment only for 100-ms CTI, *F* (6, 564) = 3.49, p < 0.01, but not for 1000-ms CTI conditions (p = 0.09).

#### PE

The error rates across the positive, neutral, and negative emotion conditions were 2.4%, 2.5%, and 2.3%, respectively, for the single block and 2.7%, 3.9%, and 2.0%, respectively, for the fade-out block. The error rates concerning the critical fade-out block were below 5% for all emotion conditions; thus, we did not pursue additional error rate analyses.

### Summary

Of the main interest, we observed that negative moods reduced the amount of RT/PE switching costs, particularly in incongruent-trial condition. On the contrary, the effect of negative moods resulted in a greater persistence of the RT fade-out costs compared to neutral and positive moods.

## Discussion

This study investigated how induced moods with different emotional valences modulate cognitive flexibility by means of a fade-out task-switching paradigm. We used emotional film clips to induce positive, neutral, and negative emotional states. The clips were retrieved from the Standard Chinese Emotional Film Clips Database^46^. The results of the subjective rating scores confirmed that we successfully induced target emotions using the clips. The study participants performed fade-out task switching so that we could investigate how emotional valence modulates cognitive flexibility on short and long time scales^24^. The switching costs and fade-out costs were evaluated as main indicators of rigidity.

The results replicated previous findings and revealed significant RT/PE switching costs^22,23^. Notably, although emotional valence did not modulate the overall RT/PE switching costs, the RT data revealed a three-way interaction of emotion, transition, and congruency, which indicated that negative moods reduced RT switching costs but only for incongruent trials. That is, RT switching costs were smaller for negative moods than neutral moods in incongruent trials.

These results are consistent with previous research suggesting that negative emotion can facilitate conflict processing^32–37^. However, the research did not specifically predict why the reduction of switching costs by negative moods occurs in only incongruent trials. The present results suggest that negative moods triggered executive control from incongruent conditions, which helped to facilitate the task-switching condition. This reasoning can be inferred from Kanske and Kotz^38^, who showed that emotional stimuli increased functional connectivity between the ventral ACC and activity in the amygdala and the dorsal ACC, which in turn correlated with facilitated executive control. Likewise, Subramaniam and colleagues^9^ suggested that positive moods would facilitate cognitive flexibility in detecting competing solution candidates via ACC activity^9^, which might imply that conflict conditions such as incongruent trials would enhance the processing of competing responses. Nevertheless, since the emotional valence investigated in these two studies^9,38^ was mainly positive, more research is still needed to test this hypothesis directly.

In contrast to negative moods, positive moods did not modulate RT/PE switching costs. This finding contrasts with some studies suggesting that the effect of positive emotion facilitates conflict processing, executive control, and creative problem solving^9,11,30,32,33,38,39^. The current results show dissociable effects of emotional valences on switching costs, which appear to be inconsistent with the theory suggesting a general mechanism for emotion (such as *appraisal theories*^40,41^) to modulate cognitive control irrespective of emotional valence.

We also found that emotional valence modulates the efficiency of disengaging from a task-switching mode to a single-task mode according to the fade-out costs. However, in contrast to the switching costs, negative moods prolonged the fade-out effect until Segment 2, but only with the short CTI of 100 ms. This is evident from the statistical results showing a significant main effect of emotion (i.e., slower RTs for negative emotion than neutral and positive emotions) in the fade-out block for only the 100-ms CTI and in Segment 2, but not for 1000-ms CTI or other segments (see Fig. 3b).

Persistence was observed in the fade-out costs associated with negative moods in relation to a faster decay of fade-out costs for neutral and positive moods. Furthermore, the difference was more pronounced in the second segment than the first segment. Thus, we suggest that negative moods do not “increase” fade-out costs but instead delay the disengagement from the previous switching mode and the transition to the current single task. This could also explain why the fade-out effect was significantly modulated by negative moods in Segment 2 rather than Segment 1.

Mayr and Liebsche^24^ developed a fade-out scenario (see also Meiran *et al*.^47^) in which a critical block condition keeps the participant ready for both tasks (a task-switching mode in a mixed-task block). However, this strategy is inappropriate because only one task is relevant in this so-called fade-out block (i.e., a single-task block), and switching no longer occurs throughout the entire fade-out block (i.e., a single-task mode). Accordingly, we suggest that negative moods cause a more persistent state that delays the disengagement from task-switching mode and the transition to a single-task mode. We suspect that negative moods narrow the scope of attention and facilitate more analytical processing, which results in a more persistent state^5,35,48,49^. Furthermore, negative moods may impede rather than facilitate the relaxation of cognitive control since fade-out costs involve the rate of disengagement from a control-demand mode (switching mode) and the transition to a less control-demand mode (single-task mode). This result also appears to be compatible with clinical findings by Meiran *et al*.^26^, who observed impaired fade-out costs for patients with unipolar depression or obsessive-compulsive disorder.

Nevertheless, some alternative explanations are also possible, such as the hypothesis of impaired working-memory updating due to negative moods. In the task-switching paradigm, goal switching requires working memory to be updated^16^, and as such, negative moods might deplete working memory resources and reduce the efficiency of updating the processing mode in working memory (task-switching mode vs. single-task mode). Another alternative explanation is that negative moods increase preparedness to switch, but that preparedness is difficult to turn off in the fade-out blocks, so it provides a bias towards preparedness to switch with negative moods. Regardless of which explanation is more feasible, the current findings of dissociable effects of emotional valences on the fade-out costs speak against the hypotheses of a general mechanism for emotion to modulate cognitive control, such as *appraisal theories*^40,41^.

The results revealed little evidence of an effect of emotion on the fade-out effect when the CTI was 1000 ms. It is unclear why the fade-out effect of emotion occurred in only the 100-ms CTI condition. One explanation might be that participants were able to return to the single-task mode easily when given more time. However, if participants really did return to the single-task mode, one would expect no further fade-out cost in the next trial since the CTI varied randomly between trials. An alternative explanation might be that emotion influences the preparation time but not the execution time, as if participants with negative emotion continue preparing for a task even though they should already be prepared. Further research is warranted to clarify this issue.

In contrast to negative moods, positive moods were not found to further modulate fade-out costs in comparison to the neutral condition. The lack of effect of positive emotions on cognitive flexibility cannot be attributed to a failure to induce positive moods since the subjective rating scales confirmed the success of inducing the targeted positive emotion. In addition, the targeted emotion induced by the film clips actually lasted throughout the task-switching session since the subjective ratings remained the same before and after the task-switching session. This suggests that the targeted emotional valence remained the same throughout the session.

Another possibility is that people generally tend to be in positive moods, so the difference between neutral and positive states may not be as obvious as between neutral and negative states. Gasper and Clore^50^ make a similar postulation. Future research could systematically manipulate different types of positive emotions (e.g., excitement/thrill vs. amusement or contentment) to determine whether there is a certain type of positive emotion that has a stronger effect on switching costs or fade-out costs.

## Conclusions

This study investigated how different emotional valences affect cognitive flexibility on short and long time scales. The results suggested that negative moods facilitate momentary switching and result in smaller switching costs, especially in incongruent trials. In addition, negative moods increased preparedness to switch, but that preparedness was difficult to turn off in the fade-out blocks, resulting in a prolonged fade-out effect. Conversely, positive moods were not found to further modulate either the short or long time scales of flexibility compared to the neutral emotional state. Therefore, the results are difficult to reconcile with the hypothesis of a general mechanism for emotion to modulate cognitive flexibility. This study contributes empirical information regarding the dissociable effects of emotional valences on the short and long time scales of cognitive flexibility.

## Method

### Ethical Statement

All of the experimental methods in this study were carried out in accordance with the Declaration of Helsinki and the rule of research in the University, and were approved by the Human Research Ethics Committee of the National Chung Cheng University, Chia-Yi, Taiwan to protect the participants’ rights. All participants signed the informed consent form before participating in the experiments.

### Participants

Forty-eight college-aged students (21 males, 27 females) ranging in age from 19 to 25 yrs (Mean = 21.15 yrs, SD = 1.77 yrs) participated in the study. Their mean years of education were 15.15 ± 1.34 yrs. All participants were right-handed, with no self-reported history of neurological or psychological disorders, and all had normal or corrected-to-normal vision. All participants were required to complete questionnaires inquiring whether they had any history of heart disease, hypertension, diabetes, brain tumor, head injury, stroke, Parkinson’s disease, or other neurological or psychiatric disorders. We conducted the experiment with the consent of each participant. Each participant was paid NT $300 (US $9) for approximately three hours of participation.

### Stimuli and Apparatus

Stimuli were presented on a 17-in monitor (resolution: 1024 × 768). E-Prime 2.0 software (Psychology Software Tools, Inc., Pittsburgh, PA), operating on an IBM-PC computer with a Pentium-4 3 G Hz processor, generated the stimuli. Participants sat approximately 100 cm from the computer screen in a sound-insulated room.

### General Procedure

The entire experiment was conducted on three separate days. At least one day inserted between sessions to reduce the carry-over effect of different emotions. Prior to the formal experimental sessions, participants reviewed and signed an informed consent form. Subsequently, participants performed the task alone in a sound-insulated room with dim lighting. Experimenters continuously observed participants via a video monitor connected with an infrared charge-coupled device camera.

In each of the three sessions, participants viewed an emotion-inducing film (positive, neutral, or negative valence; the film order was fully counterbalanced across participants), completed a task-switching paradigm, and completed the subjective emotional scales.

### Emotional Film Clips

Three emotional film clips were retrieved from the Standard Chinese Emotional Film Clips Database^46^ to induce positive (an amusing film clip), neutral (a “no emotion” film clip), or negative (a sad film clip) emotions. The duration of each film clip ranged from 3 to 5 minutes. Each clip was edited to create a coherent segment to maximize the emotional meaning of each clip. According to the normative subjective evaluation data from Liang *et al*.^46^, the mean hit rate using the scores from the emotion category scale developed by Gross and Levenson^51^ (described below) for the amusing and sad emotional film clips were approximately 90% and 91%, respectively. Hit rate refers to the percentage of participants who indicated that they had felt the target emotion at least one point more intensely than any of the other non-target emotions^51^.

### Subjective Emotion Rating Scales

Although the three film clips were constructed using the norms of the Taiwanese population, individual differences most likely exist with regard to emotions; therefore, we collected concurrent self-report data by asking participants to complete two subjective emotion rating scales after watching each film clip. Participants were required to complete one emotion dimensional scale (i.e., the Self-assessment Manikin; SAM^52,53^) and one emotion category scale^51^.

The SAM consists of three dimensions: valence, arousal, and dominance. A figure depicts values along each of these 3 dimensions using a continuous scale to indicate emotional reactions. These values range from a smiling, happy face to a frowning, unhappy face to represent the valence dimension. For the arousal dimension, the SAM ranges from an excited, wide-eyed figure to a relaxed, sleepy figure. For the dominance dimension, the SAM ranges from a large figure (in control) to a small figure (dominated). The participant can select any of the 5 figures that comprise each scale or between any two figures, thereby resulting in a 9-point scale for each dimension, where 9 represents a high rating along each dimension (i.e., high pleasure, high arousal, or high dominance), and 1 represents a low rating on each dimension (i.e., low pleasure, low arousal, or low dominance).

On the emotion category scale, participants rated the intensity of emotion that they experienced during the preceding film clip using discrete emotion terms: amusement, anger, sadness, disgust, contentment, fear, and surprise. Participants rated each emotion term with regard to intensity on 9-point Likert scales anchored by *not at all* (1) and *extremely* (9). Participants were also asked whether they looked away during the film (in which case they might not have seen important portions of the film).

### Fade-Out Task-Switching Paradigm

**Figure 4:**
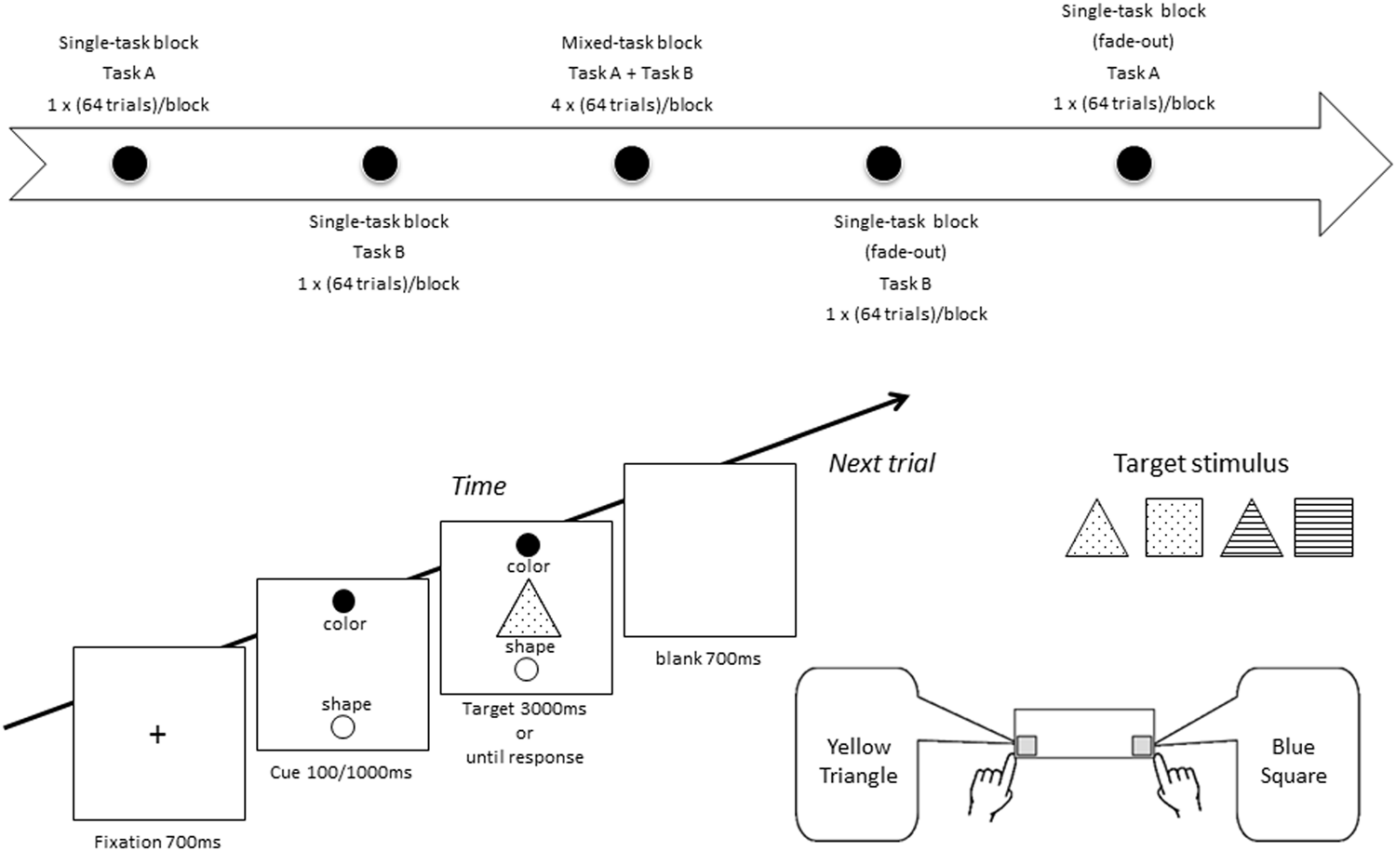
Upper panel: Block structure and procedure for a fade-out task-switching paradigm; Lower panel: Schematic illustration of a typical trial sequence, target stimulus and response mapping. Filled dots/lines in the middle triangle/square figure denote yellow/blue color.

In the fade-out task-switching paradigm, participants were cued regarding whether to respond to the color dimension (i.e., press the left key “A” for yellow and the right key “L” for blue) or the shape dimension (i.e., press the left key “A” for triangle and the right key “L” for square) of a 0.5°-x-0.5° target stimulus for the first two single-task blocks. The response key was counterbalanced between the participants. Half of the target stimuli were congruent trials in which their color dimension and shape dimension (one of the dimension was task relevant and the other was task irrelevant) were mapped to the same response key, and the other half of the stimuli were incongruent trials in which their color dimension and shape dimension were mapped to different response keys. A filled circle, presented above or below the corresponding verbal label (“Color” or “Form”), cued the response task to the target stimulus. In the first block, participants were cued to perform only one of the two tasks throughout the entire block; in the second block, participants were cued to perform the other task throughout the entire block. After these single-task blocks (their orders were counterbalanced across participants), participants were cued on a trial-by-trial basis whether to respond to the color or the shape dimension. Thus, the third block began the mixed-task blocks. Following four mixed-task blocks, participants in the critical phase (i.e., the fade-out block) were again cued to respond to only one of the two tasks throughout the entire block. There were two fade-out blocks: in the first fade-out block, participants were cued to perform only one of the two tasks throughout the entire block, and in the second fade-out block, participants were cued to perform the other task throughout the entire block. The task order of these two fade-out blocks was in reverse to that of the beginning two single-task blocks (e.g., A-B; B-A design) (see Fig. 4).

The experiment began with a warm-up block of 10 one-task trials (Color or Form), followed by a 64-trial task block. Then, a warm-up block of 10 trials of the other task was performed, following by a 64-trial block of the other task. After two single-task blocks, a warm-up block of 20 mixed-task trials appeared, followed by 4 mixed-task blocks of 64 trials each. Finally, 2 single-task blocks of 64 trials were performed. The order of the single-task blocks reversed the order at the beginning of the experiment. The order of the single-task block was counterbalanced across participants.

Each trial began following a response-cue interval of 1400 ms, including a blank screen (700 ms) and a fixation point (700 ms). This point was followed by the task cue (cue-target interval; CTI: 100 or 1000 ms, randomly varying length). Finally, the target stimulus was presented with the task cue until a response was given. A warning tone was presented for 1000 ms after an error occurred during the warm-up blocks only.

### General statistical methods

A series of repeated-measure analyses of variance (ANOVAs) were conducted using the SPSS software package (Version 18.0; SPSS Inc. Chicago, USA). We tested the effect of emotional valence on switching costs in different types of trials and different CTI conditions by performing four-way repeated-measure ANOVAs on RT and PE with within-participant independent variables of emotion (positive, neutral, and negative), transition (repeat and switch), CTI (100 ms and 1000 ms), and congruency (congruent and incongruent). We tested the effect of emotional valence on fade-out costs by performing four-way repeated-measure ANOVAs on RT and PE with independent variables of the block (single-task block and fade-out block), CTI (100 ms and 1000 ms), emotion (positive, neutral, and negative), and segment (Segment 1 through Segment 4 with 16 trials for each).

Tukey tests were performed as post-hoc analyses when a significant effect was detected for a variable with more than two levels (e.g., “emotion”). The simple main or interaction effects were determined when two or more factors showed statistically significant interactions in an ANOVA. This involves examining the effect of one factor at the level of another factor. That is, the data were split for each level of one factor, and one-way ANOVAs were performed.

As for any other one-way ANOVA with more than two levels, after a significant F is found, a post-hoc Tukey test was conducted to find out which pairs of means were statistically different. We used Bonferroni correction to adjust the p value to overcome the inflation of Type 1 error when a series of simple main effect analyses were conducted. In addition, we used the pooled error-term approach advised by Howell^54^ (pp. 483–488) for the choice of error term in the simple main effect test.

## Data Availability

The datasets generated during and/or analysed during the current study are available from the corresponding author on reasonable request.
